# Potential applications of microbial genomics in nuclear non-proliferation

**DOI:** 10.3389/fmicb.2024.1410820

**Published:** 2024-09-18

**Authors:** Heather MacGregor, Isis Fukai, Kurt Ash, Adam Paul Arkin, Terry C. Hazen

**Affiliations:** ^1^University of California, Berkeley, Berkeley, CA, United States; ^2^Bredesen Center, University of Tennessee, Knoxville, TN, United States; ^3^Department of Civil and Environmental Engineering, University of Tennessee, Knoxville, TN, United States; ^4^Department of Microbiology, University of Tennessee, Knoxville, TN, United States; ^5^Department of Earth and Planetary Sciences, University of Tennessee, Knoxville, TN, United States; ^6^Biosciences Division, Oak Ridge National Laboratory, Oak Ridge, TN, United States

**Keywords:** radionuclides, microbial genomics, nuclear non-proliferation, environmental bioindicators, microbial ecology, systems biology

## Abstract

As nuclear technology evolves in response to increased demand for diversification and decarbonization of the energy sector, new and innovative approaches are needed to effectively identify and deter the proliferation of nuclear arms, while ensuring safe development of global nuclear energy resources. Preventing the use of nuclear material and technology for unsanctioned development of nuclear weapons has been a long-standing challenge for the International Atomic Energy Agency and signatories of the Treaty on the Non-Proliferation of Nuclear Weapons. Environmental swipe sampling has proven to be an effective technique for characterizing clandestine proliferation activities within and around known locations of nuclear facilities and sites. However, limited tools and techniques exist for detecting nuclear proliferation in unknown locations beyond the boundaries of declared nuclear fuel cycle facilities, representing a critical gap in non-proliferation safeguards. Microbiomes, defined as “characteristic communities of microorganisms” found in specific habitats with distinct physical and chemical properties, can provide valuable information about the conditions and activities occurring in the surrounding environment. Microorganisms are known to inhabit radionuclide-contaminated sites, spent nuclear fuel storage pools, and cooling systems of water-cooled nuclear reactors, where they can cause radionuclide migration and corrosion of critical structures. Microbial transformation of radionuclides is a well-established process that has been documented in numerous field and laboratory studies. These studies helped to identify key bacterial taxa and microbially-mediated processes that directly and indirectly control the transformation, mobility, and fate of radionuclides in the environment. Expanding on this work, other studies have used microbial genomics integrated with machine learning models to successfully monitor and predict the occurrence of heavy metals, radionuclides, and other process wastes in the environment, indicating the potential role of nuclear activities in shaping microbial community structure and function. Results of this previous body of work suggest fundamental geochemical-microbial interactions occurring at nuclear fuel cycle facilities could give rise to microbiomes that are characteristic of nuclear activities. These microbiomes could provide valuable information for monitoring nuclear fuel cycle facilities, planning environmental sampling campaigns, and developing biosensor technology for the detection of undisclosed fuel cycle activities and proliferation concerns.

## 1 Introduction

As nuclear technology evolves in response to increased demand for decarbonization of the energy sector, new and innovative approaches are needed to effectively identify and deter the proliferation of nuclear arms, while ensuring safe, environmentally sustainable development of global nuclear energy resources (Moniz, 2019; Rockwood et al., 2019; IAEA, 2022c; Peel et al., 2022). Despite being a mature, low-carbon fuel source, concerns about nuclear weapons proliferation and environmental contamination associated with the nuclear fuel cycle (NFC) persist. Addressing these concerns is essential for maximizing the benefits of nuclear power generation (Neumann et al., 2020; Badora et al., 2021; Verma et al., 2021). Numerous field and laboratory studies have shown that prokaryotic taxa and microbially-mediated processes can, directly and indirectly, control the environmental transformation, mobility, and fate of metals and radionuclides, such as uranium, plutonium, and tritium (Lovley et al., 1991; Hazen and Tabak, 2005; Lloyd and Renshaw, 2005; Lloyd and Gadd, 2011; Renard et al., 2022; Wintenberg et al., 2023). More recently, microbial genomics integrated with machine learning models has been used to successfully predict the occurrence of heavy metals, radionuclides, and other nuclear process wastes in the environment (Smith et al., 2015; He et al., 2018; Li et al., 2020; Ghannam and Techtmann, 2021). This body of previous work provides a sound technical basis to support the use of microbial genomic data for characterizing and monitoring NFC activities.

Existing methods for monitoring proliferation activities and environmental discharges from the NFC rely on radiation detection devices and (radio) chemical analysis (IAEA, 2011). These techniques are best suited to provide precise quantitative information about specific elements and radionuclides of interest in samples of nuclear material, such as the mass of plutonium in fuel assemblies or the amount of uranium-235 on equipment surfaces in an enrichment facility (IAEA, 2022b). Microbial genomic analysis offers a complementary approach by providing insight into the biological and environmental systems interacting with released nuclides.

Microorganisms have been observed in a variety of nuclear environments, including radionuclide-contaminated sites, spent nuclear fuel pools, and cooling systems of water-cooled nuclear reactors (Rao and Nair, 1998; Francis and Dodge, 2015; Karley et al., 2018; Lopez-Fernandez et al., 2021; Barton et al., 2022; Veytskin et al., 2023). Previous studies have shed light on the diversity of microbial taxa and functional genes at various nuclear facilities and radionuclide-contaminated sites, with radiation-resistant prokaryotes such as *Deinococcus* ssp. and *Thermococcus gammatolerans* being particularly well-characterized (Daly et al., 2004; Cox and Battista, 2005; Chapon et al., 2012; Vázquez-Campos et al., 2017; Jin et al., 2019; Belykh et al., 2024). However, the mechanistic relationships underlying correlations between nuclear-related physicochemical parameters and microbial communities/functions are not well understood, and it remains unclear whether predictive relationships observed at one site can be applied to other sites exposed to similar nuclear effluent/emission streams.

This review seeks to provide a comprehensive overview of the current state of knowledge regarding microbial communities in environments exposed to nuclear effluent/emissions. Findings from this review highlight the need for data to be taken across physicochemical gradients to better identify and measure microbial responses to environmental discharges from nuclear activities, as well as the potential for leveraging global-scale datasets to enhance our understanding. By synthesizing existing research and identifying critical gaps in knowledge, this review aims to pave the way for future investigations aimed at leveraging microbial community data to predict nuclear-related physicochemical parameters in the environment. Ultimately, a deeper understanding of these complex relationships will inform more effective strategies for environmental monitoring, assessment, and remediation in nuclear-contaminated sites.

## 2 Overview of the nuclear fuel cycle and non-proliferation safeguards

### 2.1 Nuclear activities

The nuclear activities evaluated in this review include activities associated with the NFC, and nuclear legacy sites. Effluents and emissions released from these activities have distinct radionuclide signatures and physiochemical properties that could be related to microbial community structure and function (Table 1). While the focus of this review is on microbial associations with radionuclide discharges unique to nuclear industrial activities, other common wastes generated by the nuclear industry, such as heavy metals (e.g., Pb, Fe, and Zn), nitrogen compounds, acids, and volatile organic compounds, May also need to be considered when evaluating microbial community responses relevant for environmental monitoring and predictive models. Though microbial communities May exhibit sensitive and specific responses to heavy metals (e.g., total Zn, As, Pb, and U) and other non-radionuclide industrial contaminants (Boteva et al., 2016; Li et al., 2020), these waste streams are not unique to the nuclear industry. The associated microbial responses to these contaminants May therefore not be useful for distinguishing nuclear activities from other industrial activities. For example, it May be difficult to distinguish between front-end NFC activities and fertilizer production by quantifying microbial community response to total uranium, thorium, radium, or nitrogen contamination. It is also inherently difficult to evaluate the ecological effects of heavy metals separately from their individual radionuclide compositions when considering discharges from nuclear industrial activities. This is particularly true for reactor and back-end NFC environments, where most, if not all, heavy metal wastes occur in the form of actinides, fission products, and neutron activation products composed of a high percentage of synthetic radioactive nuclides that aren’t routinely measured in microbial community studies.

**Table 1:** Inventory, radioactivity (approximate order of magnitude), and physical forms of radionuclides commonly discharged to the environment during each stage of the nuclear fuel cycle.

Nuclear activity	Radionuclides^1,2,3^	Radioactivity (Bq)^2,3^	Physical forms	Other wastes^4^
Uranium mining and milling	^222^Rn; ^210^Po, ^210^Pb, ^226^Ra, ^234^Th, ^235^U, ^238^U	10^7^	Bulk solids, liquids, particulates, minor gases (e.g., radon)	Heavy metals (V, Mn, Fe, Cu, Zn, Ni, As), SO_x_ emissions, NH_3_, silica dust, acids (H_2_SO_4_)
Conversion	^222^Rn; ^210^Po, ^210^Pb, ^226^Ra, ^234^Th, ^235^U, ^238^U	10^8^	Bulk solids, liquids, minor gases and particulates	HF, F_2_, NH_3_, CaF, tributyl phosphate, kerosene
Enrichment	^222^Rn; ^99^Tc, ^210^Po, ^210^Pb, ^226^Ra, ^230^Th, ^234^Th, ^234^U, ^238^U	10^7^	Bulk solids, gases, particulates, and minor liquids	SO_x_ and NO_x_ emissions
Fabrication	^99^Tc, ^223^Ra, ^230^Th, ^232^Th, ^234^U, ^235^U, ^238^U, ^237^Np, ^241^Pu, ^241^Am	10^9^	Liquids, particulates, minor gases	HF, NH_3_, other nitrogen compounds
Reactor operations	^3^H, ^14^C, ^41^Ar, ^85^Kr, ^87^Kr, ^88^Kr, ^131^I, ^133^I, ^135^I, ^133^Xe, ^135^Xe; 51Cr, ^54^Mn, ^59^Fe, ^60^Co, ^63^Ni, ^65^Zn, ^90^Sr, ^95^Zr, ^95^Nb, ^106^Ru, ^110^Ag, ^124^Sb, ^125^Sb, ^134^Cs, ^137^Cs, ^140^Ba, ^140^La, ^144^Ce	10^12^	Gases, liquids, particulates	Minor
Interim spent fuel storage	^3^H, ^14^C, ^54^Mn, ^59^Fe, ^60^Co, ^63^Ni, ^65^Zn, ^90^Sr, ^95^Zr, ^95^Nb, ^106^Ru, ^110^Ag, ^124^Sb, ^125^Sb, ^134^Cs, ^137^Cs, ^140^Ba, ^140^La, ^144^Ce	10^10^	Liquids, minor gases	Minor
Reprocessing	^3^H, 14C, ^15^N, ^85^Kr, ^129^I, ^131^I; ^54^Mn, ^60^Co, ^63^Ni, ^65^Zn, ^90^Sr, ^95^Nb, ^95^Zr, ^99^Tc, ^106^Ru, ^110^Ag, ^125^Sb, ^134^Cs, ^137^Cs, 144Ce, ^154^Eu, ^241^Pu, ^237^Np, ^241^Am, ^242^Cm, ^244^Cm	10^15^	Gases, liquids, particulates	HCl, HNO_3_, NH_4_F NH_4_NO_3_, tributyl phosphate, kerosene

^1^List of radionuclides includes technologically-enhanced naturally occurring radioactive material (TENORM), fission products, minor actinides, neutron activation products, and decay progeny commonly discharged to the natural environment during normal operations. Nuclides are listed in order of increasing atomic mass, with common gaseous emissions listed first. Other common non-radionuclide wastes are also shown. ^2^Bq: Becquerel. ^3^Data Sources: (Costeira et al., 2020); The International Atomic Energy Agency (IAEA) database on Discharges of Radionuclides to the Atmosphere and the Aquatic Environment (DIRATA) (IAEA, 2022d; https://dirata.iaea.org) European Commission Radioactive Discharges Database (European Union, https://europa.eu/radd/index.dox). ^4^https://www.researchgate.net/profile/Velraj-Parthiban-2/publication/356774643_Transport_and_disposal_of_radioactive_wastes_in_nuclear_industry/links/61e817909a753545e2e0e9c0/Transport-and-disposal-of-radioactive-wastes-in-nuclear-industry.pdf.

The NFC encompasses the infrastructure and activities associated with the cradle-to-grave lifecycle of nuclear material (i.e., fissile material and its sources), including recovery, processing use and disposal, for purposes such as power generation, propulsion, research, or isotope production (Defense for Nuclear, 2020; IAEA, 2022e). The infrastructure and technologies used for peaceful applications of nuclear material can also be used to support the development of nuclear weapons (Defense for Nuclear, 2020; Herzog, 2020). The International Atomic Energy Agency (IAEA) is an independent, intergovernmental organization entrusted by the United Nations and the international community to deter the proliferation of nuclear weapons through the use of various tools and accounting measures referred to as safeguards (IAEA, 1970). Each stage of the fuel cycle has characteristic effluent and emission streams, which provide the basis for many monitoring technologies and environmental sampling strategies used as safeguards. These characteristic signatures and their potential microbial associations are likely to be as important for the development of biological indicators, biomonitors, or biotic indices to support future monitoring efforts for the nuclear industry. Development of effective safeguards and monitoring approaches, therefore, requires an understanding of the NFC.

**Figure 1:**
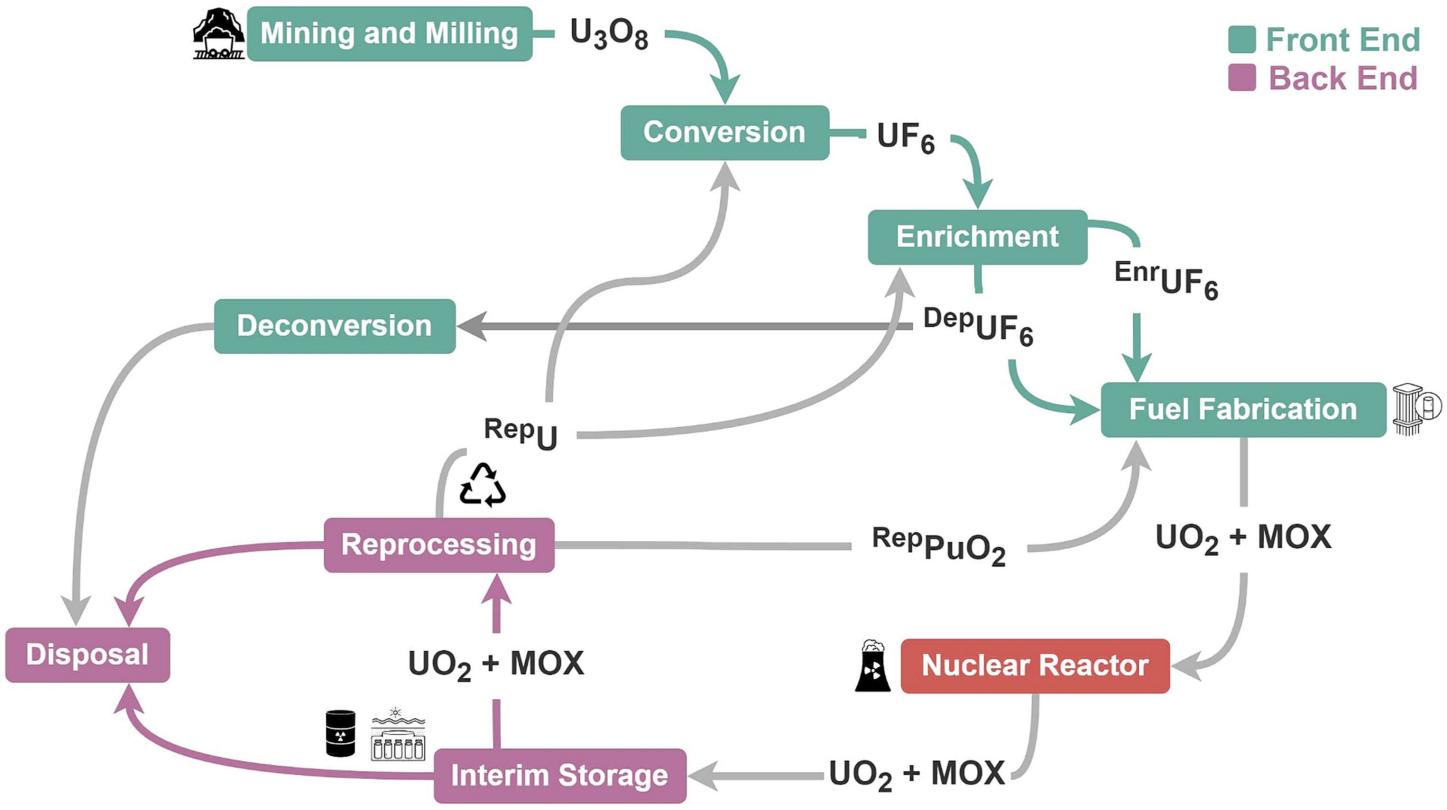
Stages of the nuclear fuel cycle.

Activities of the fuel cycle can be divided into front-end, reactor operations, and back-end stages (Figure 1), although specific components can vary depending on the technologies used, fuel types, and intended purpose, among other things (Herzog, 2020; National Academies of Science and Medicine, 2023). Front-end fuel cycle activities include uranium mining, milling, conversion, and enrichment of uranium-235 (^235^U), followed by fabrication of fuel elements for reactor use. During operation, nuclear reactors generate fission products, isotopes of plutonium, and other actinides in the fuel until a nuclear chain reaction can no longer be maintained and the fuel must be changed. Safeguard challenges for reactors include factors such as variations in reactor design, fuel types, operational schedules and frequency of fuel changes. This is particularly the case for existing research reactors, many of which use highly-enriched uranium (^235^U ≥ 20%) and require frequent fuel changes and access to hot cells where plutonium can be handled and extracted (van der Ende et al., 2019). Many novel and advanced reactor designs are expected to be deployed within the next decade, including small modular reactors, microreactors, mobile reactors, molten salt reactors, and gas-cooled reactors (Peel et al., 2022; IAEA, 2023; National Academies of Science and Medicine, 2023). Whether these new reactor designs will be more or less prone to proliferation and environmental contamination remains to be seen, but their deployment May require adjustments to the prevailing safeguards and monitoring framework for nuclear reactors.

The back end of the nuclear fuel cycle includes interim storage of spent nuclear fuel (SNF) assemblies after reactor use, followed, in some cases, by reprocessing of the fuel to extract the plutonium and uranium for reuse (closed fuel cycle). Interim storage of SNF either occurs on-site where the fuel was generated (at the reactor), or it can be transported off-site to a designated storage facility (away from the reactor) (IAEA, 2022a). Globally, 7,000 to 11,000 tons of SNF are generated annually, with approximately 20% being reprocessed for reuse and a total of 300,000 tons currently being held at interim storage facilities (IAEA, 2022e; Holdsworth et al., 2023). Commercial-scale reprocessing is currently conducted in France, India, and Russia, though smaller non-commercial, and decommissioned reprocessing facilities exist in several other countries. The final stage of the fuel cycle is the permanent disposal of SNF and high-level radioactive waste in geological repositories. Future capabilities for monitoring and characterizing nuclear material transfers will be needed as spent fuel in interim storage facilities transitions to final disposal in geological repositories. To date, only one permanent disposal facility for high-level SNF has been constructed (Finland) and is expected to begin operations before 2030 (Posiva, 2022).

An important distinction between the front-end, reactor, and back-end stages of the nuclear fuel cycle is the physiochemical nature of the waste and byproducts they produce (Table 1). Generally speaking, the radioactivity of nuclear material and associated waste increases as it moves through the fuel cycle (Costeira et al., 2020; IAEA, 2022d; National Academies of Science and Medicine, 2023). Front-end fuel cycle waste streams, such as those associated with uranium mining, milling and conversion, are typically characterized by strongly acidic or alkaline liquids, tailings, and particulates with high concentrations of metals, uranium-238 (^238^U), and ^238^U decay products [e.g., thorium-234 (^234^Th), radium-226 (^226^Ra), radon-222 (^222^Rn)]. Process material with ^235^U greater than 0.7% is generated during the enrichment stage. Environmental discharges from reactor operations reflect fission characteristics of the fuel and neutron energy in the reactor. Environmental discharges from reactor operations generally consist of atmospheric and liquid releases of noble gas radionuclides, tritium [hydrogen-3 (^3^H)], carbon-14 (^14^C), radioactive iodine species, and neutron-activated transition metals from the reactor coolant system, decontamination circuits, ventilation and emission stacks, (United States Environmental Protection, 2004; United States Nuclear Regulatory, 2021). Environmental releases from back-end fuel cycle activities are similar to those from nuclear reactors but May produce more liquid discharges containing transuranic actinides and medium- and long-lived fission products due to (re)processing, handling, and storage of spent fuel assemblies and other irradiated material (IAEA, 2022d).

### 2.2 Existing monitoring technologies for the nuclear fuel cycle

The IAEA uses several technical measures implemented through legally binding safeguard agreements to deter the spread of nuclear weapons. Examples of common safeguard activities and tools include short-notice, random, and unannounced on-site inspections, nuclear material accountancy, unattended and remote monitoring and surveillance, tamper-evident seals, nuclear material assay and analysis, and environmental sampling (IAEA, 2022f). Gamma-ray and neutron detection devices are the most commonly employed technologies for remote, continuous in-plant monitoring and on-site verification. Environmental sampling has proven to be the most useful tool for detecting clandestine nuclear material and activities at declared facilities (Donohue and Rolf, 1992; Kuhn et al., 2001; IAEA, 2011; Kemp, 2016; Schoeppner et al., 2023).

Formally designated as a safeguard in 1996, environmental sampling is routinely used to verify the absence of undeclared nuclear material and activities in and around declared sites (IAEA, 2011; Maiani et al., 2020; Schoeppner et al., 2023). ‘Location-specific’ environmental sampling is conducted during on-site inspections and generally includes collecting swipe samples from the surfaces of buildings and equipment within and immediately surrounding declared facilities to characterize the uranium and plutonium composition of material captured on the swipe, though samples of soil, water, air, and vegetation May also be collected (IAEA, 2022e). Environmental swipe samples undergo bulk and/or particle analysis off-site via techniques such as inductively coupled plasma mass spectrometry (ICP-MS), large-geometry secondary ion mass spectrometry (LG-SIMS) and scanning electron microscopy (SEM).

Wide-area environmental sampling (WAES) is another form of environmental sampling that can serve as a nuclear non-proliferation safeguard. WAES allows samples to be collected over broad areas, beyond declared facilities and sites, to evaluate the presence of undeclared nuclear materials/activities at the State- or regional-scale (IAEA, 2022e). However, early studies conducted to assess the techno-economic feasibility of regional-scale atmospheric monitoring of radiogenic gases and particulates determined WAES to be economically infeasible, and it has not yet been implemented as a safeguard by the IAEA (Wogman, 2013; Dougan et al., 2022). Advances and cost reductions in next-generation and high-throughput sequencing technologies, coupled with the development of bioinformatic and machine-learning tools, have enabled the expansion of microbial genomics into large-scale ecological assessments and environmental monitoring studies (Delgado-Baquerizo et al., 2018; Karimi et al., 2018; Cordier et al., 2019; Ghannam and Techtmann, 2021; Nayfach et al., 2021; Skidmore et al., 2022; Pavlopoulos et al., 2023). Notable projects, such as the Earth Microbiome Project and the TARA ocean project, alongside recent studies using environmental DNA/meta-barcoding, underscore the potential of microbial genomics in ecological monitoring (Sunagawa et al., 2015; Thompson et al., 2017). Measurement of taxonomic and functional diversity of soil microorganisms has also recently been proposed as part of the regulatory framework for routine monitoring of soils across the European Union (European, 2023). Publicly available data from these projects could be used to provide regional baseline data or reference databases for environmental monitoring approaches and safeguards such as WAES.

## 3 Prokaryotic microorganisms for environmental monitoring of nuclear activity

### 3.1 Importance of environmental factors

Previous research into microbial communities at sites impacted by nuclear activities has covered a plethora of facilities, including both the front- and back-end of the NFC, nuclear reactor sites, and legacy sites (Fredrickson et al., 2004; Chicote et al., 2005; Dhal et al., 2011; Vázquez-Campos et al., 2017; Nazina et al., 2020; Ihara et al., 2021; Petit et al., 2023). Legacy sites are defined as “areas contaminated by residual radioactive material deriving from past activities or events that May pose risks to health and safety or the environment, and present technical or administrative challenges to timely remediation” (IAEA, 2016).

The degree to which microorganisms are impacted by nuclear activity is dependent on the type, duration, and level of radionuclide exposure, as well as the biological, physical, and chemical parameters of the environment. Development of monitoring techniques and predictive models based on environmental microbiomes must account for the interdependencies and relative impacts of relevant environmental conditions, nuclear industrial discharges, and other anthropogenic activities (Belykh et al., 2024). Defining which parameters are relevant is key to this process, as not all factors will be important for the development of successful monitoring and modeling approaches. Constraining the sensitivity of microbial community structure and function to changes in environmental conditions and levels of radionuclide exposure is a critical step toward practical application of microbiome data in environmental monitoring.

Previous studies have shown that important environmental considerations include redox potential, pH, metals, depth, total nitrogen and organic carbon, climate, geology, and geography (Rastogi et al., 2010; Dhal and Sar, 2014; Sitte et al., 2015; Hermans et al., 2017; Coral et al., 2018; Zhu et al., 2023; Li et al., 2024). pH can be particularly crucial, as it affects the bioavailability of electron acceptors and donors, including metals, with lower pH generally increasing metal concentrations in solution (Fierer and Jackson, 2006). Total organic carbon (TOC) content is also expected to have an important effect on microbial community characteristics in any studied environment. In the context of NFC monitoring, the radioactivity of discharges released into the environmental May not be considerably different or discernable from background (per regulations), and microbial associations with radionuclide signatures May be overwhelmed by the effects of important environmental factors such as pH and TOC. This challenge can be addressed by environmental studies that integrate radionuclide analyses with routine environmental measurements and microbial genomics (Rogiers et al., 2021). For example, TOC is commonly measured in both geochemical and microbial assessments, but measurements of radiocarbon (^14^C, a common component of environmental discharges from NFC facilities) and stable carbon (^12^C, ^13^C) May also be required to the discern the potential relationship(s) between TOC, microorganisms and nuclear site activities. However, isotopic and radionuclide analyses are not commonly included in microbial ecological studies at nuclear sites. As a result, many studies May be missing a critical piece of information needed to understand how environmental factors like TOC vary between nuclear and background (non-nuclear, undeveloped or otherwise) sites, and the associated impact this has on microbial communities.

The speciation, mobility, and bioavailability of uranium is also strongly influenced by the presence and concentrations of various abiotic factors and chemical species. For example, at ambient environmental conditions, adsorption, precipitation, and ion exchange reactions between inorganic phosphate and uranium can effectively immobilize uranium and limit its interactions with microorganisms (Martinez et al., 2014). This is important to consider in environments with high phosphate levels from natural (e.g., apatite) as well as industrial and agricultural sources. Studies that carefully identify and constrain associations between environmental and anthropogenic activities have been able to effectively utilize soil bacterial community composition to predict physicochemical parameters and land use types with reasonable accuracy (Hermans et al., 2020; Lee et al., 2020).

### 3.2 Changes in microbial community diversity and taxonomy in response to nuclear activity

Studies from active and decommissioned uranium mines located in several different countries have provided important insights on microbial correlations with environmental factors and have shown that microbial communities at these sites are similar to those reported in soils with naturally elevated uranium. Soils with naturally elevated uranium levels (2,140–255,000 mg/kg) have been characterized by the presence of uncultured members of *Geobacteraceae*, *Pseudomonadaceae*, *Gallionellaceae*/*Sideroxydans*, *Holophagaceae*/*Geothrix*, and *Acidobacteria* (Mondani et al., 2011). The relative abundances of uncultured members of *Pseudomonadaceae* have also been reported to exhibit a positive correlation with uranium and ^266^Ra concentrations in groundwater downstream of a uranium deposit, alongside *Pseudomonas*, *Methylotenera*, *Planctomyces*, *Pirellula*, *Microbacterium*, and uncultured *Rhodocyclaceae*, *Saprospiraceae*, and *Sphingomonadales* (Jroundi et al., 2020).

Microbial community exposure to effluent and emissions from nuclear activity has often been associated with a decrease in alpha diversity. Sutcliffe et al. (2017) and Sutcliffe et al. (2018) investigated the microbial community diversity of natural sediments spiked with uranium (0–4,000 mg/kg) and incubated *in situ* for several months at the Ranger Uranium Mine (RUM) in Australia. Although alpha diversity metrics remained unimpacted at uranium concentrations below 4,000 mg/kg, enrichment of methanogenic archaea and several metal respiring and/or fermentative bacterial species was observed at concentrations ≥1,500 mg U/kg, with *Syntrophus*, *Geobacter*, *Dyella*, *Holophaga*, and *Geothrix*, showing notable increases along the contamination gradient (Sutcliffe et al., 2017). Another study conducted on soil samples in the RUM Land Application Areas showed that the differences in microbial communities were also associated with changes in total Ca, Al, and Cu.

Other microorganisms such as *Metallibacterium*, *Clostridium*, *Sporosarcina*, and *Sulfobacillaceae* were found to positively correlate with uranium and negatively correlate with pH in the groundwater of a decommissioned acid *in-situ* leaching (ISL) uranium mine in China (Zhu et al., 2023). In reservoir sediments downstream a uranium tailing dam, the oxidized fraction of uranium had a positive correlation with *Pseudolabrys*, *Bradyrhizobium*, and *Lacunisphaera*, while the residual fraction of uranium had a positive correlation with *Syntrophorabdus* (Liu et al., 2024).

More recently, Mallet et al. (2024) identified OTUs related to *Acidibacter*, *Acidovorax*, *Geothrix*, *Methylotenera, Geobacter*, and *Pseudolabrys* that positively correlated with concentrations of ^226^Ra, ^210^Po, and ^238^U (activity gradient of 368–1710 Bq/kg) in stream sediments impacted by an historical mining site in France. Total organic carbon content (TOC) was also reported to be a significant driver of microbial community structure at this site. Rogiers et al. (2021) investigated the microbial community of floodplain soil exposed to radionuclides from back-end NFC facilities and fertilizer production in Belgium. Positive correlations were observed between soil moisture content, *Nitrospira*, *Nitrosospira*, *Illumatobacter*, *Aridibacter,* and the activity concentrations of ^137^Cs (0.05–0.25 Bq/g), ^241^Am (0.05–0.20 Bq/g), and ^60^Co (0.005–0.012 Bq/g). However, no significant correlation was observed between these OTUs and soil pH, total organic carbon, or dissolved nitrogen (NH_4_^+^, NO_2_^−^, and NO_3_^2−^).

Prokaryotic communities have also been identified in engineered environments from nuclear reactor systems, SNF pools, and reprocessing facilities (Van Eesbeeck et al., 2021). *Sphingomonas* and *Methylobacterium* are two of the more common genera reported from engineered aqueous environments of several different nuclear reactor cooling systems and interim SNF storage facilities, where the water is routinely filtered and monitored to limit concentrations of total dissolved solids and organic content and control variation in pH, redox, and temperature. These systems provide a unique opportunity to study microbial occurrences in the presence of high radionuclide concentrations and ionizing radiation while controlling for the effects of other environmental factors encountered in natural systems.

Var*iovorax* and *Sphingomonas* were predominant in water samples from the reactor tank and primary cooling system of the Osiris nuclear research reactor (NRR) during operation (pH 7.0, *γ* activity 3,200 Bq/mL) but were replaced by *Methylobacterium*, *Asanoa*, and *Streptomyces* during shutdown (pH 5.4, γ activity 3.3 Bq/mL) (Petit et al., 2020). A similar study at the Belgian BR2 NRR reported that *Pelomonas* and *Methylobacterium* were the dominant genera during operation (pH 6 ± 0.4, γ activity 1.9 Bq/mL) and shutdown (pH 6 ± 0.4, γ activity <0.08 Bq/mL), respectively (Van Eesbeeck et al., 2021). Members of these genera, among others, have also been identified in biofilms on the walls of the water tank (pH 7.0, 143–693 Gy) of the TRIGA Mark II NRR in Slovenia (Bratkic et al., 2023).

*Acidovorax*, *Caulobacter*, and *Sphingomonas* were determined to be metabolically active and predominant members of biofilms on the walls of a SNF pool (pH 7.8, 100 Gy/h) in France (Pible et al., 2023). *Acidovorax* and *Sphingomonas* were reported in biofilms (activity 512 Bq/g) from the SNF pool of the Angra 1 nuclear power plant (NPP) in Brazil (Silva et al., 2018), and species of *Sphingomonas*, *Stenotrophomonas*, *Methylobacterium*, and *Staphylococcus*, were also isolated from a biofilm in an at-reactor interim SNF storage pool (pH 5.67, cond. 1.06 μS/cm, activity 1–500 Bq/g) at the Cofrentes NPP in Spain (Chicote et al., 2005; Sarró et al., 2005). *Staphylococcus* was present in biofilms formed under oligotrophic, high-radiation conditions at the SNF pool at the Madrid Atomic Power Station in India (Karley et al., 2019) and dominated communities from a pool and canal at the ISSF-RSG GAS facility in Indonesia (Sugoro et al., 2023). *Acidovorax*, *Sphingomonas*, and *Stenotrophomonas* were also reported in the SNF pool water (pH 11.4, activity 1,500–2000 Bq/mL, dose 5.0 × 10^4^–6.5 × 10^4^ Gy/h) of the First Generation Magnox Storage Pond at Sellafield in Cumbria, United Kingdom (Foster et al., 2020). Similarly, *Sphingomonas*, *Meiothermus*, *Methylobacterium*, and *Caulobacter* were identified in the indoor SNF pool of the Sellafield Fuel Handling Plant (pH 11.6) (Ruiz-Lopez et al., 2020). The relative abundances of OTUs associated with *Methylobacterium*, *Nitrospira*, and *Meiothermus* were significantly greater in communities from an indoor SNF disassembly basin (pH 6.1, conductivity 1.5 μS/cm) at the Savannah River Site in comparison to reference datasets (Bagwell et al., 2018).

A strain (SRS30216^T^) of *Kineococcus radiotolerans* was isolated from a floor swab taken from the inside of a hot cell containing high-level waste from plutonium-239 (^239^Pu) (re)processing at the Savannah River Site (SRS) in South Carolina (Phillips et al., 2002). Radiation levels in the hot cell were reported to range between 0.18 Gy/h to over 3.5 Gy/h (Phillips et al., 2002). The *K. radiotolerans* strain exhibited radiation and desiccation resistance similar to that of *Deinococcus radiodurans* and could respire on formic and oxalic acid present in the high-level reprocessing waste (Bagwell et al., 2008). Upon controlled, laboratory exposure to gamma radiation from a cobalt-60 source, more than 10% of *K. radiotolerans* cells were able to survive a dose of 3 kGy, nearly 1,000 times the lethal dose to humans (Phillips et al., 2002). The strain was also found to have an abundance of genes involved in DNA excision repair and detoxification of reactive oxygen species.

### 3.3 Changes in microbial community function in response to nuclear activity

Changes in the taxonomic diversity and structure of microbial communities due to exposure to nuclear discharges/activities can result in a change in the distribution of functional genes, as has been shown by Sutcliffe et al. (2017). Gene functions and metabolic pathways of microorganisms May also be restructured or abandoned as a direct effect of exposure to radionuclides and other environmental stressors. For example, increased abundance of ABC transporter genes has been observed in uranium-enriched soils (Yan et al., 2016). ABC transporters also exhibited a positive correlation with the concentration of several heavy metals, including total uranium, in a transcriptomic analysis of groundwater downgradient of a uranium deposit (Jroundi et al., 2023).

Sutcliffe et al. (2017) showed that different groups of transporters exhibited different responses to increasing concentrations of uranium. Specifically, genes involved in copper and nickel (*cbiM*, *cbiQ*, *cbiO*), zinc (*znuA*, *znuC*), and tungstate (*tupA*, *tupB*, *tupC*) uptake were enriched in highly contaminated sediments (4,000 mg U/kg), suggesting that these metals become less bioavailable at higher uranium concentrations. Similarly, phosphate transporter (*pstC*) and other genes involved in the two-component response system for phosphate limitation (*phoA*, *phoR*, *regX3*) increased with uranium concentration, while phosphonate transporters (*phnE*, *phnC*) decreased, indicating that bioavailability of phosphate ions decreases with increasing uranium levels. This is corroborated by the results of a more recent study, which reported that genes associated with phosphorus transport (*upgA*, *upgC*, *phnD*) positively correlated with the oxidizable and exchangeable fraction of uranium, while phosphorus/phosphonate transport (*phnN*, *phnK*) had an inverse relationship with total uranium (Yuan et al., 2024). These findings suggest that reduced bioavailability and biogeochemical cycling of phosphorus May be observed in environments where abiotic uranium immobilization by phosphate occurs, highlighting the importance and interdependence of biotic and abiotic factors in uranium geochemistry.

Genes involved in nitrogen and sulfur cycling have been reported to increase alongside the concentration of heavy metals and/or uranium, including those implicated in nitrogen fixation, nitrate reduction (*norB*), nitrite reduction (*nrfH*), nitrous oxide reduction (*nosZ*), and sulfate oxidation (*soxB*, *soxC*), among others (Lin et al., 2012; Sutcliffe et al., 2017; Jroundi et al., 2023; Yuan et al., 2024). However, *nirK* (copper-containing nitrate reductase) exhibited a negative correlation with total uranium, suggesting that the cytochrome c nitrate reductase *nrfH* is more effective than copper-containing nitrate reductase *nirK* in samples with higher concentrations of uranium, which May be a result of copper ions becoming less bioavailable in such environments (Sutcliffe et al., 2017; Yuan et al., 2024). Chronic exposure of groundwater microbial communities to nitrate and uranium contamination in one monitoring well at the Oak Ridge Field Research Center (ORFRC), a legacy site in Oak Ridge, Tennessee, was associated with a predominance of denitrification pathways, and abundance of genes conferring heavy metal and nitrate resistance (Hemme et al., 2010).

Meanwhile, dehydrogenase activity has been demonstrated to positively correlate with soil organic matter in uranium mining and milling-impacted soils (Kenarova et al., 2014; Boteva et al., 2016), and in one study, positively correlated with several heavy metals in addition to alkaline phosphatase activity (including Cd, Co, Cr, and Zn, but not U) (Boteva et al., 2016). These results suggest that organic matter May enable communities to overcome toxicity stress via both dehydrogenase and phosphatase activity.

### 3.4 Biosensors, bioindicators and predictive models

Many of the microorganisms and genes described above have been studied and utilized as biosensors, bioindicators and predictors of environmental contamination from NFC effluents (Supplementary Table S1). A whole-cell uranium biosensor capable of operating *in vivo* has been developed from a strain of *Caulobacter crescentus*, which fluoresces in the presence of micromolar levels of uranium (Hillson et al., 2007; Park and Taffet, 2019; Sauge-Merle et al., 2023). The *C. crescentus* biosensor can be used to discriminate between uranium contaminated (4.2 μM uranium) and uncontaminated (<0.1 μM uranium) groundwater samples (Hillson et al., 2007). More recently, a *C. crescentus* strain with improved uranium specificity was developed, but the detection limit of the biosensor remained at the micromolar level, possibly due to limited entry of uranium into the cell (Park and Taffet, 2019; Sauge-Merle et al., 2023). Strains of other genera, including *Kocuria*, *Micrococcus*, *Ochrobactrum* and *Pseudomonas*, have demonstrated bioimmobilization and bioaccumulation of radionuclides ^137^Cs and ^60^Co, highlighting their potential to serve as biosensors for radionuclides unique to the NFC (Karley et al., 2023).

*Geobacter* has been identified as playing a critical role in facilitating extracellular and interspecies electron transport in both radionuclide-contaminated and non-contaminated anaerobic environments (Cord-Ruwisch et al., 1998; Anderson et al., 2003; Lovley et al., 2011). Previous work has shown that interspecies transfer of electrons between *G. sulfurreducens* and nitrate reducing bacteria such as *Thiobacillus denitrificans* can occur via conductive nanowire appendages and nanoparticles such as magnetite of both abiotic and biotic origins (Kato et al., 2012). Because of its ecological importance in directing electron transport and coupled redox reactions in the external environment, *Geobacter* diversity and abundance along with their associated genes/gene functions, could be important bioindicators for determining the spatial and temporal extent of uranium mobility and transformation in groundwater. Other strains, including *Kocuria*, *Micrococcus*, *Ochrobactrum*, and *Pseudomonas*, have demonstrated bioimmobilization of manganese and bioaccumulation of radionuclides ^137^Cs and ^60^Co, further highlighting their potential for bioremediation and biomonitoring (Karley et al., 2023).

Other studies have used microbial genomics integrated with machine learning models to successfully predict the occurrence of heavy metals, radionuclides, and other nuclear process wastes. Smith et al. (2015) examined the potential for bacterial communities to serve as *in-situ* biosensors for the detection of groundwater contamination at the ORFRC. Using 16S rRNA amplicon sequences from 93 groundwater monitoring wells, the authors developed a Random Forest learning model that was able to accurately classify uranium-contaminated samples (F_1_ score = 0.88) based on U.S. drinking water standards. Regression analysis conducted in this study also showed statistically significant (*p* < 0.0001) correlations between 16S rRNA-predicted values and measured values of pH, manganese, lead, strontium, and 14 other geochemical parameters from the 93 groundwater wells. He et al. (2018) examined the functional diversity of groundwater microbiomes from 69 wells at the ORFRC using a functional-gene microarray (GeoChip 5.0). The overall diversity and richness of functional genes were reported to decrease with increased uranium concentrations. The exceptions to this were *dsrA* genes/gene variants from *Halorthodospira, Desulfobulbus, Pelodictyon*, and *Vibrio* species, which were observed to increase significantly (*p* < 0.05) with increased uranium. Higher levels of uranium in groundwater were also associated with increased abundances of cytochrome and hydrogenase genes from *Geobacter, Dechloromonas, Enterobacter, Pseudomonas, Alcaligenes, Desulfovibrio, Desulfitobacterium, Rhodobacter, Ochrobactrum*, and *Anaeromyxobacter*. Analysis of functional responses to nitrate gradients showed significant (*p* < 0.05) increases in the abundance of *nirk* genes from fungi, uncultured bacteria and *Pseudomonas* and *napA* genes from *Beggiatoa*, *Vibrio*, *Campylobacter*, and *Dinoroseobacter* species with increased nitrate concentrations. The predictive power of these and other gene families/categories was evaluated via the use of random forest algorithms for feature selection, classification/regression and estimation of error rate. Results showed that 50 key functional genes related to uranium reduction could be used to identify uranium contaminated wells with an out-of-bag prediction error of 11.5%. Similarly, fifty-four genes involved in nitrogen cycling were used to predict the occurrence of nitrate contaminated wells at an estimated error of 15.94%.

The potential for microbial genes and gene functions to be used as biosensors, bioindicators and predictors of NFC effluent is also supported by results of transcriptomic and metabolomic studies. Laboratory experiments by Wintenberg et al. (2023) examined the transcriptional response of *Escherichia coli* after one and 15 days of continuous exposure to ionizing radiation from ^239^Pu, ^3^H, and ^55^Fe at a constant dose rate of ~10 mGy/day, based on United States Department of Energy guidelines for environmental dose limits (Energy USDO, 2015). Differential expression analysis revealed that unique transcriptional responses from this model bacterium could effectively be used to discriminate between short- and long-term exposures to alpha decay, beta decay and gamma radiation from the three different radionuclides. Results of this and other similar studies suggest that exposure to radionuclides of biologically relevant elements, such as ^3^H and short-lived neutron-activated transition metals, could have a measurable effect on prokaryotic microorganisms (Renard et al., 2022; Kolesnik et al., 2023; Lai et al., 2023; Wintenberg et al., 2023).

## 4 Discussion

Constraining the sensitivity of microbial community structure and function to changes in environmental conditions and levels of radionuclide exposure is a critical step toward practical application of microbiome data in environmental monitoring. Radiological releases during routine operation of nuclear facilities are designed to be sufficiently low so as to not pose immediate danger to human health and macrofauna, but previous work described in this review suggests the impact of these releases on environmental microbiomes could be measurable. The distribution and fate of radionuclides released into the environment from nuclear activities/sites, including whether or not they can be recovered in sufficient quantity and quality for analysis, partially depends on environmental factors such as climate, redox conditions, pH, and of course, microbial activity (Carvalho et al., 2023). Land management practices related to aquaculture, agriculture, and other industries have been shown to significantly shape the composition of soil bacterial communities, and some studies have been able to effectively utilize these relationships to predict physicochemical parameters and land use types with reasonable accuracy (Hermans et al., 2017; Delgado-Baquerizo et al., 2018; Cordier et al., 2021). It has been shown that soil bacterial communities exhibit more variance across varying environmental parameters than across different climates or increasing geographic distance. The recent expansion of microbial genomics into large-scale ecological assessments and environmental monitoring studies has provided large publicly available databases that could be used to establish regional baseline/reference microbial community conditions needed for development of microbial-based environmental monitoring techniques for the nuclear industry.

**Figure 2:**
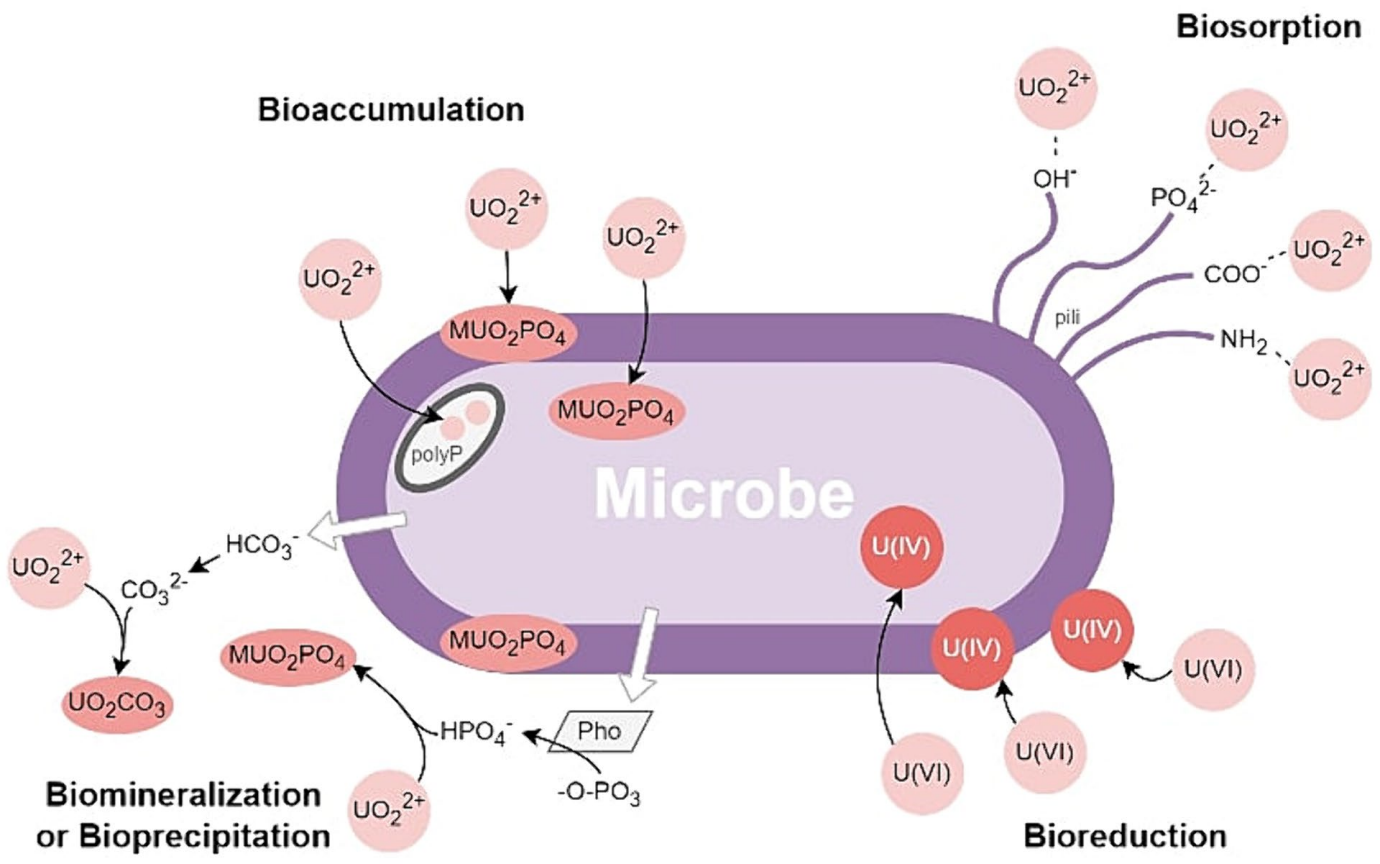
Microbially induced Uranium Biomineralization or Bioprecipitation, Bioreduction, Biosorption, and Bioaccumulation.

Existing studies provide valuable insights into the mechanisms underlying correlations between nuclear-related physicochemical parameters and microbial communities/functions. It’s now understood that microbial physiology and functions can, directly and indirectly, influence the mobility and stability of uranium and plutonium phases through mechanisms such as adsorption, bio-oxidation/reduction, intracellular accumulation, biomineralization, and bio-precipitation (see Figure 2; Macaskie and Basnakova, 1998; John et al., 2001; Cardenas et al., 2008; Francis and Dodge, 2015; Wufuer et al., 2017; Ren et al., 2022). In addition to their potential use as biosensors/bioindicators, the ability of archaea and bacteria to immobilize and concentrate uranium and radionuclides could also have useful applications for contaminant monitoring and mitigation in the nuclear industry.

Data from existing environmental studies have helped to identify microbial-geochemical associations in environments exposed to NFC effluent, with microbial signatures of uranium contamination identified in several of the studies reviewed. However, information needed to effectively evaluate associations with radiological factors at a given site is somewhat lacking. Total uranium and bulk metal concentrations are often presented as signatures of nuclear contamination without information on the radionuclide compositions of the sampled nuclear environment or contemporaneous measurements from a representative background area. This information is critical for validating nuclear contamination signatures and establishing a link between the observed microbial-geochemical associations and site activities/effluent.

Nuclear legacy sites have arguably provided the most extensive data on the microbial ecology of natural environments impacted by nuclear activities. Studies from these sites have demonstrated the potential use of microbial genomics for predicting uranium and nitrate contamination in groundwater systems, with sulfate-reducing bacteria (*Geobacter*, *Desulfovibrio*), denitrifying bacteria (*Pseudomonas*, *Thiobacillus denitrificans*) and their associated genes (*nosZ*, *nirk*, *dsrA, cytochrome-C and hydrogenases*) being identified as key players in a number of studies (Istok et al., 2004; Hemme et al., 2010; Lin et al., 2012; He et al., 2018). In contrast to legacy sites, there are few publicly available studies describing the microbial, geochemical and radiological characteristics of natural environments surrounding non-legacy nuclear facilities with normal operational histories. These sites, which represent the majority of existing nuclear facilities, need to be studied in order to validate and constrain signatures of exposure in natural environments, and help to ensure legacy sites are not overrepresented in our data/models.

Studies that have explored the relationships between microbial community structure/function and physicochemical characteristics of effluent from nuclear activities are largely site-specific and the results are difficult to compare across sites. While site- and niche-specific factors are extremely important for assessing microbial community responses, discharges from nuclear activities, anthropogenic land use, and environmental conditions (e.g., temperature, rainfall, soil geochemistry) have regional as well as site-specific characteristics. For example, elevated ^14^C has been found in terrestrial environments 5 km away from nuclear power plants in both France and Brazil (Roussel-Debet et al., 2006; Dias et al., 2008). Site-specific analysis might not capture important processes at these distances. To develop monitoring approaches that can be applied to many sites and across large areas, such as for WAES, regional assessments could help identify commonalities and more widespread phenomena that explain how local-scale microbial-radionuclide interactions translate into macro-scale observations.

The focus of many microbial community analyses in radionuclide-contaminated environments has been on identifying and characterizing the most abundant, resilient, and/or culturable taxa, especially those that have potential for bioremediation. Some similarities are observed in the microbial communities reported from engineered aqueous nuclear environments, such as reactor tanks/pools and interim SNF storage pools, with *Sphingomonas*, *Methylobacterium*, *Meiothermus*, *Acidovorax*, and *Nitrospira* being commonly reported in these environments (Chicote et al., 2005; Sarró et al., 2005; Bagwell et al., 2018; Petit et al., 2020; Foster et al., 2023). Further work needs to be done to identify and enumerate taxa that are characteristic and common to these, and other environments exposed to effluent from nuclear-related activities. Currently available sequencing data from nuclear sites are largely amplicon sequences, and measurements that provide deeper biological and (radio) chemical information are also needed. Additional studies integrating metagenomic and/or transcriptomic analyses with representative radiological data/conditions (Lai et al., 2023; Wintenberg et al., 2023) would help to constrain the sensitivity and reproducibility of microbial-radionuclide interactions from field- and model-based observations.

In order to accurately predict physicochemical parameters, higher-resolution models that include a wider array of well-ordered sampling and categorization of nuclear sites, their effluent signatures, and representative background sites are needed to help validate contamination signatures and determine whether predictive relationships observed at one site can be applied to other sites exposed to similar nuclear effluent/emission streams. Acquiring all the available (meta) data from a site can be challenging due to the sensitive and sometimes proprietary nature of nuclear-related technologies and activities. Inconsistent and/or incomplete data acquisition and reporting can impede the utility of machine learning models for identification and interpretation of predictive relationships. It is critical to work with well-annotated data to ensure information is properly integrated and compared across different datasets from different sources. Encoding associated environmental data, such as latitude/longitude coordinates, concentrations of chemical species, temperature, pH, and other relevant environmental parameters, enables better comparability across studies (Cernava et al., 2022). Adopting data reporting standards such as MIxS (Minimum Information about any (x) Sequence) is also vital for cross-study comparisons and meta-analyses of environmental sequence data (Yilmaz et al., 2011). With these practices in place, identification of true biological signals and correction/removal of technical or noise-related variations will be improved, helping researchers understand potential sources of variation and make more informed interpretations of the results. Well-labeled and standardized datasets will also allow machine learning tools to more effectively parse, analyze, and contextualize the data in relation to other studies and existing literature.

## 5 Conclusion

Microbial analysis has the potential to extend nuclear nonproliferation safeguards and monitoring capabilities to natural environments outside of facilities. Incorporation of environmental microbiome analysis into large-scale ecological assessments and environmental monitoring studies, along with advances and cost reductions in sequencing technologies, provides a potential avenue for development of technically and economically feasible environmental monitoring safeguards such as WAES. Due to the complexity and heterogeneity of environmental systems, a combination of techniques and multiple lines of evidence will be needed to help inform safeguards conclusions and contaminant mitigation strategies for a growing nuclear industry. The benefit of microbial data is that it can serve as a qualitative indicator of radionuclide discharges derived from operational activities of nuclear facilities, including undeclared activities/locations, facility leaks, and emerging contaminant issues that May otherwise go undetected. The use of microbial data as a predictive tool to screen for radionuclide contamination and provide semiquantitative constrains on environmental conditions (e.g., pH, temperature, redox, C:N) could help optimize the collection, analysis and interpretation of data for quantitative (radio)chemical analyses. Additional research is needed to quantify and constrain microbial responses to specific signals of nuclear activity, understand the long-term resilience of these microbial indicators, and develop standardized protocols for environmental sampling and data analysis. This research will help establish the reliability and robustness of microbial data as a complementary tool in nuclear environmental monitoring and safeguard strategies, ultimately enhancing the ability to detect and mitigate nuclear contamination in various environmental contexts.
